# IR voices about COVID 19

**DOI:** 10.1186/s42155-020-00136-z

**Published:** 2020-07-07

**Authors:** Robert Morgan, Mohammad Arabi, Yasuaki Arai, Marco Das, Jafar Golzarian, Andrew Holden, Shuvro H. Roy-Choudhury, Stavros Spiliopoulos, Gao-Jun Teng, Maria Tsitskari

**Affiliations:** 1 St George’s University of London, Blackshaw Road, London, SW17 0QT UK; 2 Riyadh, Saudi Arabia; 3 National Cancer Center, Tokyo, Japan; 4 Helios Hospital Duisberg, Duisberg, Germany; 5 University of Minnesota, Minneapolis, USA; 6 Auckland City Hospital, Auckland, New Zealand; 7 NH Group of Hospitals, Kolkata, India; 8 Attikon University General Hospital, Athens, Greece; 9 Zhongda Hospital, South East University, Nanjing, China; 10 Apollonion Hospital, Nicosia, Cyprus

The COVID 19 pandemic has had a major impact on healthcare and healthcare resources. It is well-known that medical personnel working on the treatment frontline, such as pulmonologists, internists, intensive care and emergency care staff have worked extensively to cope with the challenges of COVID while being exposed to great risks of infection themselves. Interventional radiology (IR) is a profession that connects with many other hospital specialties and has a long tradition of adapting well to new situations. Naturally, this is dependent on local circumstances and may vary from country to country. CVIR Endovascular would like to understand how interventional radiology and interventional radiologists have been affected by this pandemic. We invited several IRs from around the world to discuss their personal experiences of the COVID 19 pandemic, and their comments are summarised below.

## What was the effect of COVID 19 on hospitals?

In general, this depended on several factors: large vs small hospitals, public vs private institutions, general vs specialist hospitals. In particular, the designation of a hospital as a COVID centre i.e. accepting admissions of COVID 19 patients via the emergency department and referrals from other hospitals had a major effect on IR departments working in these hospitals.

Whichever type of hospital, most institutions significantly changed their way of working. To manage the opposing demands of positive/suspected COVID 19 patients versus non COVID 19 patients and the protection of hospital staff and the public, major changes were undertaken in standard operating procedures. Many hospitals divided up into COVID positive and negative areas. In many cases staff were also divided up into those working in COVID areas and non COVID areas and were not allowed to cross over from one area to the other, either on the same day or in some cases weeks at a time. All medical leave was cancelled in most hospitals, although as most countries were in Lockdown, there was nowhere to go, even if leave could be taken.

The topic of Personal Protective Equipment (PPE) and the lack of adequate supply affected many hospitals in many countries. Similarly, the relative lack of intensive care (ICU) facilities and ventilators were a worldwide phenomenon.

## What was the effect of COVID 19 on interventional radiology departments?

All IR departments wherever they were located have been affected by the COVID pandemic. Most IR departments had to make major changes to their operating procedures in terms of infection control, new IR rotas, and the case mix of IR cases undertaken. Reassuringly, IR played a major part in the hospital approach to the pandemic in many hospitals.

All IRs reported that elective cases were cancelled or postponed, and the focus of IR departments changed radically to help hospitals cope with the large numbers of COVID patients. IRs reported that peripheral vascular disease cases vanished from many hospitals. Similarly interventional oncology cases such as percutaneous tumour ablation and TACE were cancelled or postponed.

## Did it change your way of working?

Many hospitals with large IR departments divided the IR staff into teams working in separate rotas. One benefit of these teams or Pods was that if one team member caught COVID 19, then only the team was affected and not the whole IR department.

Many departments divided their IR rooms into “clean rooms” vs “dirty rooms” treating COVID and non COVID patients in separate environments. This was inevitably a challenge for smaller IR departments.

IRs performing procedures on COVID positive and suspected COVID patients had to wear full PPE. Wearing full PPE required elaborate and time-consuming donning (putting on) and doffing (taking off) procedures. Moreover, IR rooms required extensive cleaning and disinfecting in between procedures. The combination of PPE and room-cleaning generally increased the time taken to perform procedures and reduced the number of procedures that could be performed per day. In general, there were less cases which took longer.

An additional disadvantage of PPE is that it is hot and uncomfortable for the wearer. This is increasingly so for longer procedures and some IRs reported that it had the potential to impact on their performance.

Many IRs reported that one of the positive aspects to arise out of the pandemic was that clinic consultations had become virtual or online in almost all cases. It seems likely that virtual clinics will be with us long after COVID 19 has gone.

## Did IR contribute to COVID 19 treatment?

In general, IR played an important role in the treatment of severely ill patients with COVID 19 infection. Many IR teams volunteered to provide a “lines insertion service” for the central venous and arterial lines on the ICU and at the bedside on the COVID wards. All of these procedures undertaken in unfamiliar surroundings away from the IR department were performed in full PPE, which added to the challenge. Many pleural and abdominal drainage procedures were performed at the bedside away from the usual location in IR departments. The main caseload of many IR departments during the pandemic surge consisted mainly of central venous line insertions, drainage procedures, and feeding tube placements including gastrostomy tubes.

## How did COVID 19 affect you personally?

Several IRs were concerned that elective cases had to be cancelled or postponed and the effects that this had on their patients. Understandably, the rapid changes brought about by the pandemic were associated with fear and stress, changing to relief after passage of the COVID peaks in their individual countries. However, most IRs reported feelings of satisfaction and pride at providing an important service for their patients and their hospitals.

In the absence of live educational events, online educational webinars and discourse with other IRs was generally appreciated. All IRs reported that they enjoyed travelling less, working from home occasionally and spending more time with their families.

## Summary

The COVID 19 pandemic has impacted to a substantial degree on interventional radiology practice and interventional radiologists around the world. Despite the challenges that COVID 19 has produced, Interventional Radiologists have once again risen to the challenge to help their patients and their hospital colleagues. This has been, and will continue to be, a major area for engagement, participation and research by interventional radiologists around the world.

## Data Availability

Not applicable.

